# Association between Polymorphisms in the Renin-Angiotensin-Aldosterone System Genes and Essential Hypertension in the Han Chinese Population

**DOI:** 10.1371/journal.pone.0072701

**Published:** 2013-08-28

**Authors:** Lindan Ji, Xiaobo Cai, Lina Zhang, Lijuan Fei, Lin Wang, Jia Su, Lissy Lazar, Jin Xu, Yaping Zhang

**Affiliations:** 1 Department of Biochemistry, School of Medicine, Ningbo University, Ningbo, China; 2 State Key Laboratory of Genetic Resources and Evolution, Kunming Institute of Zoology, Chinese Academy of Sciences, Kunming, China; 3 Department of Preventive Medicine, School of Medicine, Ningbo University, Ningbo, China; 4 Department of Pathology, School of Medicine, Ningbo University, Ningbo, China; 5 Department of Cardiology, The Affiliated Ningbo No.1 Hospital, School of Medicine, Ningbo University, Ningbo, China; Kunming Institute of Zoology, Chinese Academy of Sciences, China

## Abstract

**Background:**

Renin-angiotensin-aldosterone system (RAAS) is the most important endocrine blood pressure control mechanism in our body, genes encoding components of this system have been strong candidates for the investigation of the genetic basis of hypertension. However, previous studies mainly focused on limited polymorphisms, thus we carried out a case-control study in the Han Chinese population to systemically investigate the association between polymorphisms in the RAAS genes and essential hypertension.

**Methods:**

905 essential hypertensive cases and 905 normotensive controls were recruited based on stringent inclusion and exclusion criteria. All 41 tagSNPs within RAAS genes were retrieved from HapMap, and the genotyping was performed using the GenomeLab SNPstream Genotyping System. Logistic regression analysis, Multifactor dimensionality reduction (MDR), stratified analysis and crossover analysis were used to identify and characterize interactions among the SNPs and the non-genetic factors.

**Results:**

Serum levels of total cholesterol (TC) and triglyceride (TG), and body mass index (BMI) were significantly higher in the hypertensive group than in the control group. Of 41 SNPs genotyped, rs3789678 and rs2493132 within *AGT*, rs4305 within *ACE*, rs275645 within *AGTR1*, rs3802230 and rs10086846 within *CYP11B2* were shown to associate with hypertension. The MDR analysis demonstrated that the interaction between BMI and rs4305 increased the susceptibility to hypertension. Crossover analysis and stratified analysis further indicated that BMI has a major effect, and rs4305 has a minor effect.

**Conclusion:**

These novel findings indicated that together with non-genetic factors, these genetic variants in the RAAS may play an important role in determining an individual’s susceptibility to hypertension in the Han Chinese.

## Introduction

Essential hypertension, defined as high blood pressure (BP) with no identifiable cause, affecting 95% of hypertensive patients [1]. It is considered to be the consequence of an interaction between environmental and genetic factors [2]. Hitherto, many candidate genes in the renin-angiotensin-aldosterone system (RAAS), the sympathetic nervous system, and water-sodium balance system have been widely studied [3]–[6]. Among all these genes which play important roles in the etiology of hypertension, those encoding the main components of the RAAS are deemed the most possible candidate genes since the RAAS plays a fundamental role in the maintenance of blood pressure and cardiovascular homeostasis [7], [8].

RAAS genes encoding renin (*REN*), angiotensinogen (*AGT*), angiotensin-converting enzyme (*ACE*), angiotensin type 1 receptor (*AGTR1*) and aldosterone synthase gene (*CYP11B2*) have been widely investigated in different ethnic populations, and dozens of single nucleotide polymorphisms (SNPs) within RAAS genes have been reported to be significantly associated with essential hypertension [9]–[11]. However, previous studies focused on limited SNPs like *AGT* M235T, *AGT* A6G, *AGT* T174M, *ACE* I/D, *AGTR1* A1166C, and *CYP11B2* C3344T, and the results are often inconsistent [12]–[15]. Moreover, dozens of genome-wide association studies (GWAS) on hypertension have been carried out, but none of these hot polymorphisms was significantly associated with hypertension [16]–[19]. It is possible that environmental factors, population variation, relatively small sample size, patient selection, and limited genetic alleles may contribute to the conflicting or even contradictory results [20]–[22].

Given these limitations, this study was designed and conducted in a large, homogeneous sample of Han Chinese, which would minimize the potential influence of mixed factors on hypertension. The objective of the present study was to systemically examine the association between polymorphisms in the RAAS candidate genes (*REN*, *AGT*, *ACE*, *AGTR*, and *CYP11B2*) and hypertension. Thus, we first conducted a case-control study in the Han Chinese population and genotyped all tagSNPs within RAAS genes. Subsequently, we analyze the interaction among different SNPs and non-genetic risk factors for hypertension, which may give more information on the roles of genetic factors.

## Materials and Methods

The protocol of this study was reviewed and approved by the medical ethics committee of Ningbo University. The health records and blood samples of the participants were collected with informed written consent.

### Study Participants

The participants were chosen from our established community-based epidemiologic study of common diseases. With informed written consent, we collected more than 10,000 health records. Subsequently, participants who fulfilled the following criteria were put into our database: 30 to 75 years old, Han Chinese, living in Ningbo City (East coast of China) for at least three generations without migration history. Finally, 905 essential hypertensive cases and 905 normotensive controls were chosen from this database, which were matched for age and sex. In addition, participants without cardiovascular diseases, diabetes, kidney diseases, or other major chronic illnesses according to their health records were recruited as controls.

### Measurement of Blood Pressure and Clinical Parameters

Blood pressure was measured in the morning after the participants had been in sitting position for 10 minutes. Three readings were taken 5 minutes apart using standard mercury sphygmomanometer and the average of last two measurements was recorded. Hypertension in this study was defined as sitting systolic blood pressure (SBP) ≥140 mmHg and/or diastolic blood pressure (DBP) ≥90 mmHg, or self-reported use of anti-hypertensive medication. Patients with secondary hypertension were excluded. Normal blood pressure was defined with SBP≤120 mmHg and DBP≤80 mmHg.

With informed written consent, two milliliter of venous blood was collected with ethylene diamine tetraacetic acid (EDTA) as anticoagulant. Subsequently, serum levels of total cholesterol (TC), high-density lipoprotein (HDL), triglyceride (TG) were measured enzymatically on a Hitachi automatic biochemistry analyzer 7100. Clinical information including body mass index (BMI), and weekly alcohol and cigarettes consumption were also obtained. In this study, who consumed ≥70 g of alcohol per week for more than 1 year were defined as individuals with alcohol abuse. Moreover, who smoked ≥70 cigarettes per week for more than 1 year were defined as individuals with smoking habit.

### SNP Genotyping

All 41 tagSNPs were retrieved from HapMap (http://hapmap.ncbi.nlm.nih.gov/), with tagger pairwise method in CHB: R^2^ cut off = 0.8 and minor allele frequency (MAF) cut off = 0.1 (Table S1). The genomic DNA was extracted from the whole blood using standard phenol-chloroform extraction method. Genotyping was performed using the GenomeLab SNPstream Genotyping System (Beckman Coulter Inc.) according to the manufacturer’s protocol [23].

### Statistical Analysis

Continuous variables were presented as the mean ± SD and analyzed by t-test between two groups. Statistical analysis of allele and genotype frequencies between case and control groups were compared by chi-squared test. Effect of confounding variables were identified by logistic regression (SPSS 16.0, SPSS Inc.). Hardy-Weinberg equilibrium (HWE) was calculated in controls by the software PEDSTATS V0.6.8 (http://www.sph.umich.edu/csg/abecasis/). Multifactor dimensionality reduction (MDR), stratified analysis and crossover analysis were used to identify and characterize interactions among the SNPs and the non-genetic factors [24]. The software used for MDR is distributed in a JAVA platform with a graphical user interface and is freely available online (http://www.epistasis.org/mdr.html).

All tests were two-sided, and *P* values less than 0.05 were considered statistically significant. For chi-squared test, the *P* values were adjusted for the total number of tested SNPs using the Bonferroni correction method (α = 0.05/41 ≈ 0.0012).

## Results

The baseline characteristics of our study participants are summarized in Table 1. The male to female ratio was equal in both groups, and mean age of hypertensive participants and controls were similar, demonstrating that the hypertensive and control groups were well-matched and are appropriate for the following analyses. Serum high-density lipoprotein (HDL) and percentage of regular smoking and alcohol abuse showed no difference between hypertensive and control groups. However, serum levels of TG and TC, and BMI were significantly higher in the hypertensive groups than in the control group (*P*<0.01).

**Table 1:** Baseline characters of the investigated participants.

Variables	Case	Control	*P*-value
Number	905	905	
Male/Female	392/513	392/513	
Age (y)	56.91±7.37	56.60±7.51	*P* = 0.38
BMI(Kg/m^2^)	24.65±3.24	23.21±2.86	*P*<0.01
HDL (mM)	1.41±0.35	1.41±0.32	*P* = 0.72
TC (mM)	5.34±1.00	5.17±0.93	*P*<0.01
TG (mM)	2.02±1.68	1.63±1.12	*P*<0.01
Smoking habit	173	147	*P* = 0.11
Alcohol abuse	152	148	*P* = 0.80

BMI, body mass index; HDL, high-density lipoprotein; TC, total cholesterol; TG, triglyceride.

**Figure 1:**
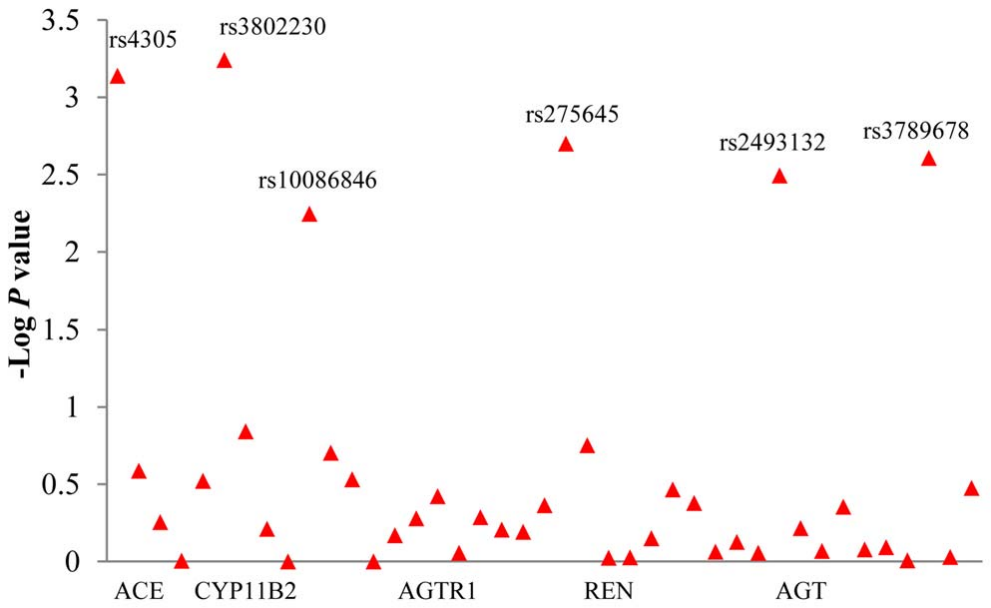
Negative Log *P* values for the association of 41 single-nucleotide polymorphisms in 5 candidate genes of renin-angiotensin-aldosterone system with hypertension. The *P* values were obtained from the comparison of two allele frequencies. Labeled SNPs had a *P* value less than 0.01.

The *P* value of 41 tagSNPs within RAAS genes were shown in Figure 1. The rs10935724 within *AGTR1* and rs6414 within *CYP11B2* were failed in genotyping, and the genotyping success rate of other 39 SNPs was 99%. Two of 39 SNPs, rs3789678 and rs10086846, deviated from Hardy-Weinberg equilibrium (*P*<0.05). However, the genotype distribution and MAF of these two SNPs were similar to those of HapMap CHB. To exclude the genotyping error, we randomly regenotyped 20% of the samples for these two SNPs by Tm-shift genotyping method [25], the results were same. Therefore, they were still included in the following analyses. In addition, with the prevalence, odds ratio (OR), and MAF in this study, the Genetic Power Calculator (available online http://pngu.mgh.harvard.edu/~purcell/gpc/) indicated that the sample size is big enough to do case-control analysis with 80% power [26]. According to the chi-square test *P* values (*P*<0.05) and odds ratios, rs3789678 and rs2493132 within *AGT*, rs4305 within *ACE*, rs275645 within *AGTR1*, rs3802230 and rs10086846 within *CYP11B2* were shown to associate with hypertension (Table 2). No significant association was found between polymorphisms within *REN* and hypertension. The genotype information for the remaining 35 SNPs that did not reach significance in the association analyses were shown in Table S2. After Bonferroni correction, only rs4305 and rs3802230 were still significant, the other 4 SNPs were marginally significant.

**Table 2:** Genotype distributions of those SNPs significantly associated with hypertension.

SNP	Gene	Group	Genotype			MAF	*P* Value	OR	95% CI
rs3789678	*AGT*	Case	690 (CC)	159 (CT)	45 (TT)	0.14	0.002	1.32	1.10–1.58
		Control	626 (CC)	236 (CT)	41 (TT)	0.18			
rs2493132	*AGT*	Case	373 (CC)	410 (CT)	111 (TT)	0.35	0.003	1.23	1.07–1.40
		Control	316 (CC)	452 (CT)	137 (TT)	0.40			
rs4305	*ACE*	Case	155 (AA)	441 (AG)	300 (GG)	0.36	0.001	1.26	1.10–1.44
		Control	103 (AA)	450 (AG)	348 (GG)	0.42			
rs275645	*AGTR1*	Case	455 (AA)	395 (AG)	43 (GG)	0.27	0.002	1.27	1.09–1.48
		Control	535 (AA)	333 (AG)	37 (GG)	0.22			
rs3802230	*CYP11B2*	Case	80 (AA)	348 (AC)	470 (CC)	0.28	0.001	1.28	1.13–1.48
		Control	89 (AA)	428 (AC)	385 (CC)	0.34			
rs10086846	*CYP11B2*	Case	469 (CC)	277 (CT)	151 (TT)	0.32	0.006	1.21	1.06–1.39
		Control	413(CC)	318 (CT)	172 (TT)	0.37			

The *P* values were obtained from the comparison of two allele frequencies. OR, odds ratio; CI, confidence interval.

Considering the effect of confounding variables, we further carried out logistic regression analysis with genetic and non-genetic factors. The result showed that rs2493132, rs10086846, and TC were no longer associated with hypertension (*P*>0.05, Table 3).

**Table 3:** Logistic regression for genetic and non-genetic factors.

Variables	B	*P* value	Exp (B)	95% CI
rs3789678	0.216	**0.033** *	1.241	1.02–1.51
rs2493132	0.131	0.108	1.140	0.97–1.34
rs4305	0.242	**0.001** *	1.273	1.10–1.47
rs275645	0.276	**0.001** *	1.318	1.11–1.56
rs3802230	0.313	**0.011** *	1.368	1.08–1.74
rs10086846	0.063	0.551	1.065	0.87–1.31
BMI	0.143	**0.000** *	1.154	1.12–1.19
TC	0.077	0.149	1.080	0.97–1.20
TG	0.138	**0.001** *	1.148	1.06–1.25
Constant	−5.432	**0.000** *	0.004	

The *P* values of 6 SNPs were obtained from the comparison of two allele frequencies. BMI, body mass index; TC, total cholesterol; TG, triglyceride.

* *P* value was less than 0.05.

Moreover, MDR was used to analyze the interaction among ‘significant’ SNPs and non-genetic risk factors for hypertension. After input the genotypes of 6 SNPs together with information about TG, TC, and BMI, the software output the best model for ‘BMI and rs4305’ with 10/10 Cross-validation Consistency (Table 4). In order to delineate how BMI and rs4305 interacts to cause hypertension, we carried out crossover analysis. The result showed that both BMI and the A allele of rs4305 increased the susceptibility to hypertension, but BMI had the main effect (Table 5). The stratified analysis further showed that when BMI ≥25, the A allele of rs4305 has no association with hypertension [*P = *0.85, OR = 1.02, 95% confidence interval (CI) = 0.81–1.30] (Table 6). However, when BMI <25, the A allele showed significant association with hypertension (*P*<0.001, OR = 1.41, 95% CI = 1.19–1.66) (Table 6), which also indicates that BMI has the major effect, and rs4305 had a minor effect.

**Table 4:** MDR analysis of gene-environment interaction.

Best model	TestingAccuracy	Testing Sensitivity	Testing Odds Ratio	Testing X^2^	Cross-validation Consistency
BMI	0.59	0.43	2.26 (95%CI: 1.20–4.27)	6.50 (*P* = 0.011)	10/10
BMI, rs4305	0.60	0.53	2.32 (95%CI: 1.27–4.23)	7.62 (*P* = 0.006)	10/10
BMI, rs275645, rs3802230	0.57	0.53	1.82 (95%CI: 1.01–3.29)	3.94 (*P* = 0.047)	5/10

**Table 5:** Crossover analysis of interaction between BMI and rs4305.

BMI	Allele	Case	Control	*P* Value	OR	95% CI
<25	G	581	876	1	1	NA
≥25	A	300	172	<0.001	2.63	2.12–3.26
≥25	G	460	270	<0.001	2.57	2.14–3.09
<25	A	451	484	<0.001	1.41	1.19–1.66

The *P* values were obtained from the comparison of two allele frequencies. OR, odds ratio; CI, confidence interval.

**Table 6:** Stratified analysis of interaction between BMI and rs4305.

BMI	Group	GG	GA	AA	G	A	*P* Value	OR	95% CI
<25	Case	160	261	95	581	451	*P*<0.001	1.41	1.19–1.66
	Control	271	334	75	876	484			
≥25	Case	140	180	60	460	300	*P* = 0.85	1.02	0.81–1.30
	Control	77	116	28	270	172			

The *P* values were obtained from the comparison of two allele frequencies. OR, odds ratio; CI, confidence interval.

## Discussion

Since RAAS is the most important mechanism regulates blood pressure in our body [27], genes encoding components of this system have been strong candidates for the investigation of the genetic basis of hypertension and major targets for antihypertensive drugs [28]. However, previous studies mainly focused on limited polymorphisms, thus we carried out a case-control study to systemically investigate the association between polymorphisms in the RAAS genes and essential hypertension. The present study identified several novel genetic variants in the RAAS genes that may play critical roles in BP regulation and susceptibility for hypertension. According to Chi-square test and logistic regression analysis, rs3789678 within *AGT*, rs4305 within *ACE*, rs275645 within *AGTR1*, rs3802230 within *CYP11B2* were shown to associate with hypertension.

Similar to this study, some of the susceptibility SNPs were also found to be associated with hypertensive traits in previous studies. The rs4305 has been related to the risk of hypertension (*P = *3.0×10^−5^), and associated with SBP (*P = *4.6×10^−4^) and DBP (*P = *6.0×10^−5^) in a study compromising 23 cohorts of three independent studies [Cohorts for Heart and Aging Research in Genomic Epidemiology Consortium (CHARGE); Global BPgen Consortium; and Women’s Genome Health Study] and a total of 86,588 participants [29]. Furthermore, a recent GWAS for ACE enzyme activity found strong association of rs4343 with increased activity in the Han Chinese (*P = *3.0×10^−25^), and the mean ACE activity among subjects with G allele of rs4343 increased by 3.5 IU/L per copy of the allele [30]. The G allele of rs4343 is in strong linkage disequilibrium (LD) with A allele of rs4305 (HapMap JPT/CHB D’ = 0.97, r^2^ = 0.80), suggesting a potential common link between the studies. In the present study, A allele of rs4305 increases the susceptibility to hypertension, which might be associated with increased ACE activity.

Prior to our study, Chen *et al*. also studied the association of *CYP11B2* gene and essential hypertension in southwest Han Chinese population [31]. Four tag SNPs (rs4536, rs4545, rs3097, and 3802230) within the *CYP11B2* gene were selected through HapMap. In addition, C344T (rs1799998) and K173R (rs4539) polymorphisms that previous studies were mostly interested, were also selected for the study. The result showed that among the six SNPs, only the C allele of rs3802230 was significantly more prevalent in the hypertension subjects than in the control subjects (*P = *0.006, OR = 1.28, 95% CI: 1.07–1.52). Since the results of both studies were similar, we further calculated the pooled *P* value and OR. The combined *P = *0.001, OR = 1.20, 95% CI = 1.08–1.34, I^2^ = 0.0% (*P = *0.39), which means no heterogeneity existed between two studies, and the C allele of rs3802230 might be a risk factor for essential hypertension in the Han Chinese population. Chen *et al.* also analyzed these SNPs in Yi and Hani Minorities of China, and found rs4536 was significantly associated with hypertension in the Hani minority, however, no association was found in the Yi minority [32].

Pickering and colleagues have initially suggested that hypertension and blood pressure are complex traits [33], and previous epidemiologic studies have found dozens of risk factors, such as obesity, high-fat diets, smoking, alcohol abuse, excessive salt intake, mental stress, and others to associate with high blood pressure [34]–[36]. There is growing evidence that interactions among multiple genes and environmental factors may play an important role in determining the susceptibility to various common diseases including hypertension [37]. Our previous study indicated that interaction analysis might give a little more information than the single genetic study [38]. In the present study, high BMI and serum TG level were confirmed as risk factors for hypertension by logistic regression analysis. The MDR analysis further demonstrated that the interaction between BMI and rs4305 was associated with hypertension. Since BMI represents the internal metabolic and physiological environment that plays a key role in development of high blood pressure [39], and ACE is one of the most important target for design of anti-hypertensive drugs, it’s not surprising that the interaction of them may play an important role in the susceptibility to hypertension. Previous genetic epidemiologic study also found the interactions between *MMP3* gene polymorphism rs679620 and BMI in predicting blood pressure in African-American women with hypertension [40]. The recent important genetic studies are mainly carried out in well-organized cohorts like Global BPgen, CHARGE, and GenSalt Study, which means the epidemiologic data are readily available [19], [41]–[43]. With the development of statistic methods for evaluation of gene-environment interaction, we can expect more missing inheritability to be found [44], [45].

In conclusion, we identified several genetic variants in the RAAS genes that were significantly associated with hypertension in the Han Chinese population. Most notable, the interaction between BMI and rs4305 increased the susceptibility to hypertension, meanwhile BMI has a major effect, and rs4305 has a minor effect.

## Supporting Information

Table S1 **All 41 tagSNPs within genes coding for RAAS.**
(DOC)

Table S2 **Genotype distributions of 35 SNPs not associated with hypertension.**
(DOC)
