# Supplementary material for: Association between Polymorphisms in the Renin-Angiotensin-Aldosterone System Genes and Essential Hypertension in the Han Chinese Population
Source: PLoS One. 2013 Aug 28;8(8):e72701. doi: 10.1371/journal.pone.0072701 (PMC3756014; doi:10.1371/journal.pone.0072701)
Supplement: Table S1 — All 41 tagSNPs within genes coding for RAAS. (DOC) [file pone.0072701.s001.doc]

Table S1 All 41 tagSNPs within genes coding for RAAS

| **Gene** | **tagSNP** |
| --- | --- |
| *ACE* | rs4305, rs4329, rs4353, rs4461142, rs4968591 |
| *AGT* | rs7536290, rs11568046, rs11122575, rs2478523, rs2493132, rs7079, rs3789671, rs2478543, rs11122577, rs3889728, rs1078499, rs7539020, rs3789678, rs2493137, rs4628514 |
| *AGTR1* | rs4681157, rs2933249, rs10935724, rs931490, rs3772616, rs12721241, rs275649, rs6801836, rs1800766, rs5182, rs2675511, rs275645, rs275643 |
| *CYP11B2* | rs3802230, rs6433, rs4545, rs6414, rs10086846 |
| *REN* | rs1464816, rs11571080, rs11571078 |
