# Supplementary material for: Association between Polymorphisms in the Renin-Angiotensin-Aldosterone System Genes and Essential Hypertension in the Han Chinese Population
Source: PLoS One. 2013 Aug 28;8(8):e72701. doi: 10.1371/journal.pone.0072701 (PMC3756014; doi:10.1371/journal.pone.0072701)
Supplement: Table S2 — Genotype distributions of 35 SNPs not associated with hypertension. (DOC) [file pone.0072701.s002.doc]

Table S2 Genotype distributions of 35 SNPs not associated with hypertension

| **SNP** | **Gene** | **Genotype** | ***P* Value** | **OR** | **95%CI** |
| --- | --- | --- | --- | --- | --- |
| rs11122575 | *AGT* | Case: 117 (CC), 420 (CT), 357 (TT) Control: 120 (CC), 412 (CT), 372 (TT) | 0.75 | 1.02 | 0.89-1.17 |
| rs2478543 | *AGT* | Case: 33 (AA), 271 (AG), 592 (GG) Control: 36 (AA), 272 (AG), 595 (GG) | 0.85 | 0.98 | 0.83-1.16 |
| rs1078499 | *AGT* | Case: 67 (CC), 356 (CT), 470 (TT) Control: 68 (CC), 354 (CT), 483 (TT) | 0.81 | 1.02 | 0.88-1.18 |
| rs3789671 | *AGT* | Case 272 (AA) 449 (AC) 177 (CC) Control 264 (AA) 454 (AC) 184 (CC) | 0.61 | 1.04 | 0.91-1.18 |
| rs7539020 | *AGT* | Case: 80 (CC), 390 (CT), 423 (TT) Control: 85 (CC), 388 (CT), 432 (TT) | 0.98 | 1.00 | 0.87-1.15 |
| rs7079 | *AGT* | Case: 18 (AA), 232 (AC), 649 (CC) Control: 21 (AA), 229 (AC), 647 (CC) | 0.87 | 0.98 | 0.82-1.18 |
| rs3889728 | *AGT* | Case: 234 (AA), 453 (AG), 210 (GG) Control: 238 (AA), 445 (AG), 220 (GG) | 0.84 | 1.01 | 0.89-1.16 |
| rs11122577 | *AGT* | Case: 24 (AA) , 281 (AC), 590 (CC) Control: 31 (AA), 285 (AC), 579 (CC) | 0.44 | 0.94 | 0.79-1.11 |
| rs2478523 | *AGT* | Case: 178 (CC), 446 (CT), 265 (TT) Control: 193 (CC), 435 (CT), 277 (TT) | 0.88 | 0.99 | 0.87-1.13 |
| rs11568046 | *AGT* | Case: 10 (CC), 223 (CT), 657 (TT) Control: 9 (CC), 211 (CT), 679 (TT) | 0.42 | 1.08 | 0.89-1.32 |
| rs2493137 | *AGT* | Case: 355 (CC), 410 (CT), 126 (TT) Control: 357 (CC), 426 (CT), 122 (TT) | 0.93 | 0.99 | 0.87-1.14 |
| rs7536290 | *AGT* | Case: 574 (AA), 285 (AG), 39 (GG) Control: 555 (AA), 311 (AG), 38 (GG) | 0.34 | 1.08 | 0.92-1.27 |
| rs4628514 | *AGT* | Case: 214 (CC), 455 (CT), 232 (TT) Control: 194 (CC), 467 (CT), 241 (TT) | 0.33 | 1.07 | 0.94-1.22 |
| rs4353 | *ACE* | Case: 322 (CC), 443 (CT), 129 (TT) Control: 322 (CC), 439 (CT), 144 (TT) | 0.56 | 1.04 | 0.91-1.19 |
| rs4461142 | *ACE* | Case: 298 (CC), 441 (CT), 159 (TT) Control: 299 (CC), 444 (CT), 159 (TT) | 0.99 | 1.00 | 0.88-1.14 |
| rs4329 | *ACE* | Case: 100 (AA), 408 (AG), 388 (GG) Control: 112 (AA), 419 (AG), 369 (GG) | 0.26 | 0.92 | 0.81-1.06 |
| rs4968591 | *ACE* | Case: 43 (CC), 303 (CT), 536 (TT) Control: 28 (CC), 312 (CT), 552 (TT) | 0.30 | 1.09 | 0.93-1.28 |
| rs12721241 | *AGTR1* | Case: 15 (AA), 180 (AG), 705 (GG) Control: 19 (AA), 190 (AG), 694 (GG) | 0.38 | 0.91 | 0.75-1.12 |
| rs931490 | *AGTR1* | Case: 745 (AA), 143 (AG), 5 (GG) Control: 751 (AA), 144 (AG), 9 (GG) | 0.68 | 1.05 | 0.83-1.32 |
| rs10935724 | *AGTR1* | NA | | | |
| rs1800766 | *AGTR1* | Case: 27 (CC), 266 (CT), 602 (TT) Control: 30 (CC), 274 (CT), 598 (TT) | 0.62 | 0.96 | 0.81-1.14 |
| rs5182 | *AGTR1* | Case: 83 (CC), 384 (CT), 430 (TT) Control: 83 (CC), 373 (CT), 444 (TT) | 0.64 | 1.03 | 0.90-1.19 |
| rs2675511 | *AGTR1* | Case: 22 (CC), 283 (CT), 595 (TT) Control: 30 (CC), 249 (CT), 621 (TT) | 0.43 | 1.07 | 0.90-1.27 |
| rs2933249 | *AGTR1* | Case: 680 (CC), 204 (CT), 14 (TT) Control: 705 (CC), 187 (CT), 13 (TT) | 0.29 | 0.90 | 0.74-1.10 |
| rs275649 | *AGTR1* | Case: 4 (AA), 192 (AG), 695 (GG) Control: 11 (AA), 184 (AG), 710 (GG) | 0.88 | 0.98 | 0.80-1.21 |
| rs6801836 | *AGTR1* | Case: 13 (CC), 202 (CT), 686 (TT) Control: 16 (CC), 208 (CT), 673 (TT) | 0.52 | 0.94 | 0.77-1.14 |
| rs3772616 | *AGTR1* | Case: 19 (AA), 303 (AG), 571 (GG) Control: 46 (AA), 268 (AG), 589 (GG) | 0.53 | 0.95 | 0.80-1.12 |
| rs275643 | *AGTR1* | Case: 673 (AA), 211 (AG), 13 (GG) Control: 700 (AA), 196 (AG), 8 (GG) | 0.18 | 0.87 | 0.72-1.06 |
| rs4681157 | *AGTR1* | Case: 40 (AA), 299 (AC), 560 (CC) Control: 30 (AA), 288 (AC), 581 (CC) | 0.20 | 1.11 | 0.95-1.31 |
| rs6433 | *CYP11B2* | Case: 631 (AA), 245 (AG), 22 (GG) Control: 665 (AA), 217 (AG), 21 (GG) | 0.14 | 1.15 | 0.96-1.37 |
| rs4545 | *CYP11B2* | Case: 247 (AA), 453 (AG), 200 (GG) Control: 246 (AA), 442 (AG), 214 (GG) | 0.62 | 1.03 | 0.91-1.18 |
| rs6414 | *CYP11B2* | NA | | | |
| rs11571080 | *REN* | Case: 233 (AA), 451 (AG), 208 (GG) Control: 228 (AA), 470 (AG), 205 (GG) | 0.94 | 1.01 | 0.88-1.15 |
| rs11571078 | *REN* | Case: 667 (CC), 210 (CT), 19 (TT) Control: 664 (CC), 215 (CT), 21 (TT) | 0.71 | 1.04 | 0.86-1.25 |
| rs1464816 | *REN* | Case: 51 (AA), 323 (AC), 525 (CC) Control: 39 (AA), 340 (AC), 509 (CC) | 0.94 | 1.01 | 0.86-1.17 |
